# Supplementary material for: Characterization of Two Complete Mitochondrial Genomes of Atkinsoniella (Hemiptera: Cicadellidae: Cicadellinae) and the Phylogenetic Implications
Source: Insects. 2021 Apr 11;12(4):338. doi: 10.3390/insects12040338 (PMC8070250; doi:10.3390/insects12040338)
Supplement: Supplementary file 1 [file insects-12-00338-s001.zip › Supplementary Materials/new version Table S1.docx]

**The starting partitions used to initiate the PartitionFinder analysis.**

| **Dataset** | **starting partitions** |
| --- | --- |
| AA | ATP6 = 1-170;  ATP8 = 171-183;  COX1 = 184-688;  COX2 = 689-898;  COX3 = 899-1155;  CYTB = 1156-1515;  ND1 = 1516-1707;  ND2 = 1708-1925;  ND3 = 1926-1988;  ND4 = 1989-2320;  ND4L = 2321-2384;  ND5 = 2385-2809;  ND6 = 2810-2890; |
| PCG12 | ATP6_pos1 = 1-426\2;  ATP6_pos2 = 2-426\2;  ATP8_pos1 = 427-500\2;  ATP8_pos2 = 428-500\2;  COX1_pos1 = 501-1516\2;  COX1_pos2 = 502-1516\2;  COX2_pos1 = 1517-1964\2;  COX2_pos2 = 1518-1964\2;  COX3_pos1 = 1965-2480\2;  COX3_pos2 = 1966-2480\2;  CYTB_pos1 = 2481-3236\2;  CYTB_pos2 = 2482-3236\2;  ND1_pos1 = 3237-3852\2;  ND1_pos2 = 3238-3852\2;  ND2_pos1 = 3853-4452\2;  ND2_pos2 = 3854-4452\2;  ND3_pos1 = 4453-4672\2;  ND3_pos2 = 4454-4672\2;  ND4_pos1 = 4673-5496\2;  ND4_pos2 = 4674-5496\2;  ND4L_pos1 = 5497-5666\2;  ND4L_pos2 = 5498-5666\2;  ND5_pos1 = 5667-6738\2;  ND5_pos2 = 5668-6738\2;  ND6_pos1 = 6739-7024\2;  ND6_pos2 = 6740-7024\2; |
| PCG12RNA | ATP6_pos1 = 1-426\2;  ATP6_pos2 = 2-426\2;  ATP8_pos1 = 427-500\2;  ATP8_pos2 = 428-500\2;  COX1_pos1 = 501-1516\2;  COX1_pos2 = 502-1516\2;  COX2_pos1 = 1517-1964\2;  COX2_pos2 = 1518-1964\2;  COX3_pos1 = 1965-2480\2;  COX3_pos2 = 1966-2480\2;  CYTB_pos1 = 2481-3236\2;  CYTB_pos2 = 2482-3236\2;  ND1_pos1 = 3237-3852\2;  ND1_pos2 = 3238-3852\2;  ND2_pos1 = 3853-4452\2;  ND2_pos2 = 3854-4452\2;  ND3_pos1 = 4453-4672\2;  ND3_pos2 = 4454-4672\2;  ND4_pos1 = 4673-5496\2;  ND4_pos2 = 4674-5496\2;  ND4L_pos1 = 5497-5666\2;  ND4L_pos2 = 5498-5666\2;  ND5_pos1 = 5667-6738\2;  ND5_pos2 = 5668-6738\2;  ND6_pos1 = 6739-7024\2;  ND6_pos2 = 6740-7024\2;  16S = 7025-8034;  12S = 8035-8651; |
