# Supplementary material for: Characterization of Two Complete Mitochondrial Genomes of Atkinsoniella (Hemiptera: Cicadellidae: Cicadellinae) and the Phylogenetic Implications
Source: Insects. 2021 Apr 11;12(4):338. doi: 10.3390/insects12040338 (PMC8070250; doi:10.3390/insects12040338)
Supplement: Supplementary file 1 [file insects-12-00338-s001.zip › Supplementary Materials/new version Table S2.docx]

Table S1 The Start codons and stop codons of each protein coding gene in nine Cicadellinae mitogenomes

| Gene | Start Codon/Stop Codon | | | | | | | | |
| --- | --- | --- | --- | --- | --- | --- | --- | --- | --- |
|  | *Atkinsoniella grahami* | *Atkinsoniella xanthonota* | *Homalodisca vitripennis* | *Bothrogonia ferruginea* | *Cuerna* sp. | *Cicadella viridis* (KY752061) | *Cicadella viridis* (MK335936) | *Cofana yasumatsui* | *Bothrogonia qiongana* |
| ND2 | ATT/TAA | ATT/TAA | ATT/TAA | ATT/TAA | ATT/TAA | ATT/TAA | ATT/TAA | ATA/TAA | ATT/TAA |
| COX1 | ATG/TAA | ATG/TAA | ATG/T | ATG/TAG | ATG/TAA | ATG/TAA | ATG/TAA | ATG/TAA | ATG/TAG |
| COX2 | ATT/T | ATT/T | ATT/T | ATA/T | ATT/T | ATT/TA | ATT/T | ATT/TA | ATT/T |
| ATP8 | TTG/TAA | TTG/TAG | TTG/TAG | ATA/TAA | TTG/TAA | ATA/TAA | ATA/TAA | TTG/TAA | ATA/TAA |
| ATP6 | ATG/TAA | ATG/TAA | ATG/TAA | ATA/TAA | ATG/TAA | ATG/TAA | ATA/TAA | ATA/TAG | ATA/TAA |
| COX3 | ATG/TAA | ATG/TAA | ATG/TAA | ATG/TAA | ATG/TAA | ATG/T | ATG/T | ATG/TAA | ATG/TAA |
| ND3 | ATT/TAA | ATT/TAA | ATA/TAG | ATT/T | ATA/TAA | ATC/TAA | ATT/TAG | ATA/TAG | ATT/TAG |
| ND5 | ATT/TAA | ATT/TAA | ATT/T | ATT/TAA | TTG/T | TTG/T | TTG/T | TTG/T | TTG/TAA |
| ND4 | ATG/TAA | ATG/TAA | ATG/TAA | ATA/TA | ATG/TAA | ATG/TAA | ATA/TAA | ATA/TAA | ATA/TAA |
| ND4L | ATG/TAA | ATG/TAA | ATG/TAA | ATG/TAA | ATG/TAA | ATG/TAA | ATG/TAA | ATG/TAA | ATG/TAA |
| ND6 | ATT/TAA | ATT/TAA | ATT/TAA | ATA/TAA | ATA/TAA | ATT/TAA | ATT/TAA | ATC/TAA | ATA/TAA |
| CYTB | ATG/TAG | ATG/TAA | ATG/TAG | ATG/TA | ATG/TAG | ATG/TAA | ATG/TAG | ATG/TAG | ATG/TAA |
| ND1 | ATT/TAA | ATT/TAA | ATT/TAA | ATG/TAA | ATT/TAA | ATT/TAA | ATT/TAA | ATT/TAA | ATG/TAA |

* The codons on the left and right of slashes (/) are the start codons and stop codons, respectively.
