# Supplementary material for: Characterization of Two Complete Mitochondrial Genomes of Atkinsoniella (Hemiptera: Cicadellidae: Cicadellinae) and the Phylogenetic Implications
Source: Insects. 2021 Apr 11;12(4):338. doi: 10.3390/insects12040338 (PMC8070250; doi:10.3390/insects12040338)
Supplement: Supplementary file 1 [file insects-12-00338-s001.zip › Supplementary Materials/new version Table S3.docx]

Table S1 Partition strategies and evolutionary models of AA, PCG12 and PCG12RNA datasets used in the phylogenetic analysis

|  | Subset | Best Model | Sites | Partition names |
| --- | --- | --- | --- | --- |
| AA | 1 | MTART+I+G+F | 1372 | COX2, CYTB, ATP8, COX3, ND2, ND6, ATP6, ND3 |
|  | 2 | MTART+I+G | 505 | COX1 |
|  | 3 | MTART+I+G+F | 1013 | ND1, ND5, ND4L, ND4 |
| PCG12 | 1 | GTR+I+G | 213 | ATP6_pos1 |
|  | 2 | TVM+I+G | 213 | ATP6_pos2 |
|  | 3 | TVM+I+G | 37 | ATP8_pos1 |
|  | 4 | GTR+I+G | 37 | ATP8_pos2 |
|  | 5 | GTR+I+G | 508 | COX1_pos1 |
|  | 6 | TVM+I+G | 508 | COX1_pos2 |
|  | 7 | GTR+I+G | 224 | COX2_pos1 |
|  | 8 | TVM+I+G | 224 | COX2_pos2 |
|  | 9 | GTR+I+G | 258 | COX3_pos1 |
|  | 10 | TVM+I+G | 258 | COX3_pos2 |
|  | 11 | GTR+I+G | 378 | CYTB_pos1 |
|  | 12 | GTR+I+G | 378 | CYTB_pos2 |
|  | 13 | TVM+I+G | 308 | ND1_pos1 |
|  | 14 | TVM+I+G | 393 | ND1_pos2, ND4L_pos2 |
|  | 15 | GTR+I+G | 410 | ND2_pos1, ND3_pos1 |
|  | 16 | TVM+I+G | 300 | ND2_pos2 |
|  | 17 | GTR+I+G | 253 | ND6_pos2, ND3_pos2 |
|  | 18 | TVM+I+G | 412 | ND4_pos1 |
|  | 19 | GTR+I+G | 412 | ND4_pos2 |
|  | 20 | GTR+I+G | 621 | ND5_pos1, ND4L_pos1 |
|  | 21 | GTR+I+G | 536 | ND5_pos2 |
|  | 22 | TIM+I+G | 143 | ND6_pos1 |
| PCG12RNA | 1 | GTR+I+G | 213 | ATP6_pos1 |
|  | 2 | TVM+I+G | 356 | ATP6_pos2, ND6_pos2 |
|  | 3 | TVM+I+G | 37 | ATP8_pos1 |
|  | 4 | GTR+I+G | 37 | ATP8_pos2 |
|  | 5 | GTR+I+G | 508 | COX1_pos1 |
|  | 6 | TVM+I+G | 508 | COX1_pos2 |
|  | 7 | GTR+I+G | 224 | COX2_pos1 |
|  | 8 | GTR+I+G | 602 | CYTB_pos2, COX2_pos2 |
|  | 9 | GTR+I+G | 258 | COX3_pos1 |
|  | 10 | TVM+I+G | 258 | COX3_pos2 |
|  | 11 | GTR+I+G | 378 | CYTB_pos1 |
|  | 12 | GTR+I+G | 308 | ND1_pos1 |
|  | 13 | GTR+I+G | 308 | ND1_pos2 |
|  | 14 | GTR+I+G | 410 | ND2_pos1, ND3_pos1 |
|  | 15 | TVM+I+G | 300 | ND2_pos2 |
|  | 16 | GTR+G | 110 | ND3_pos2 |
|  | 17 | TVM+I+G | 412 | ND4_pos1 |
|  | 18 | GTR+I+G | 497 | ND4L_pos2, ND4_pos2 |
|  | 19 | GTR+I+G | 85 | ND4L_pos1 |
|  | 20 | GTR+I+G | 536 | ND5_pos1 |
|  | 21 | GTR+I+G | 536 | ND5_pos2 |
|  | 22 | TIM+I+G | 143 | ND6_pos1 |
|  | 23 | GTR+I+G | 1627 | 16S, 12S |

AA: amino acid sequences of the protein-coding genes (PCGs) from 73 species; PCG12: first and second codon positions of PCGs from 73 species; PCG12RNA, the first and the second codon positions of the PCGs and two rRNA genes from 67 species.
